# Giant traumatic pancreatic pseudocyst in a 17-year-old male: a case report

**DOI:** 10.1093/jscr/rjag203

**Published:** 2026-03-29

**Authors:** Radisnay G Lambert, Barbara Y H Cervantes, Mariuska R Gonzalez, Fernando M A Acevedo, Duniesky M Lopez

**Affiliations:** Department of Surgery, School of Medicine UHAS, PMB 31 Ho, Ghana; Department of Surgery, School of Medicine UHAS, PMB 31 Ho, Ghana; Department of Surgery, School of Medicine UHAS, PMB 31 Ho, Ghana; Department of Dentistry, School of Medicine UHAS, PMB 31 Ho, Volta region, Ghana; Department of Internal Medicine, School of Medicine UHAS, Volta region, PMB 31 Ho, Ghana

**Keywords:** pancreatic pseudocyst, abdominal trauma, cystogastrostomy, adolescent

## Abstract

Pancreatic pseudocysts are localized fluid collections surrounded by non-epithelialized walls, typically arising as a complication of pancreatitis or pancreatic trauma. While common in adults with a history of pancreatitis, traumatic pancreatic pseudocysts in adolescents are rare. This case report describes an unusual presentation of a giant traumatic pancreatic pseudocyst in a 17-year-old male following abdominal trauma. The case highlights the diagnostic challenges, surgical management, and postoperative course of this condition in a young patient. By presenting this unique case, we aim to contribute to the limited literature on traumatic pancreatic pseudocysts in adolescents and provide insights into effective management strategies in resource-limited settings. This case report describes a 17-year-old male who developed a giant pancreatic pseudocyst following abdominal trauma from a bicycle accident. The patient presented with abdominal distention and pain. Diagnostic imaging revealed an 18.5 × 10.0 × 13.5 cm pancreatic pseudocyst. Management involved open transgastric cystogastrostomy, resulting in successful drainage. Despite a postoperative surgical site infection, the patient achieved full recovery. This case highlights the importance of considering pancreatic pseudocysts in young trauma patients and demonstrates the efficacy of surgical management in resource-limited settings.

## Introduction

Pancreatic pseudocysts are localized fluid collections surrounded by fibrous or granulation tissue, typically arising as a complication of acute or chronic pancreatitis, or less commonly, pancreatic trauma [1]. These lesions occur in ~10%–20% of patients with acute pancreatitis and 20%–40% of those with chronic pancreatitis [2]. While pancreatic pseudocysts are well-documented in adults, their occurrence in children and adolescents is relatively rare, with traumatic etiology being even less common [3].

The management of pancreatic pseudocysts has evolved significantly over the past decades, with treatment options ranging from conservative approaches to minimally invasive interventions and surgical procedures [4]. The choice of treatment depends on various factors, including the size and location of the pseudocyst, its duration, and the presence of complications [5]. In recent years, endoscopic drainage has gained popularity due to its minimally invasive nature and favorable outcomes [6]. However, in certain cases, particularly those involving large or complex pseudocysts, surgical intervention may still be necessary [7].

This case report presents an unusual instance of a giant traumatic pancreatic pseudocyst highlighting the diagnostic challenges, complex surgical management, and postoperative course associated with this condition.

## Case presentation

A 17-year-old male student was presented after being referred with complaints of abdominal distention and pain. The patient had no family history of chronic diseases or personal history of comorbidities. The only significant history was a severe trauma to the upper abdomen following a bicycle accident ~3 months prior to presentation.

**Figure 1:**
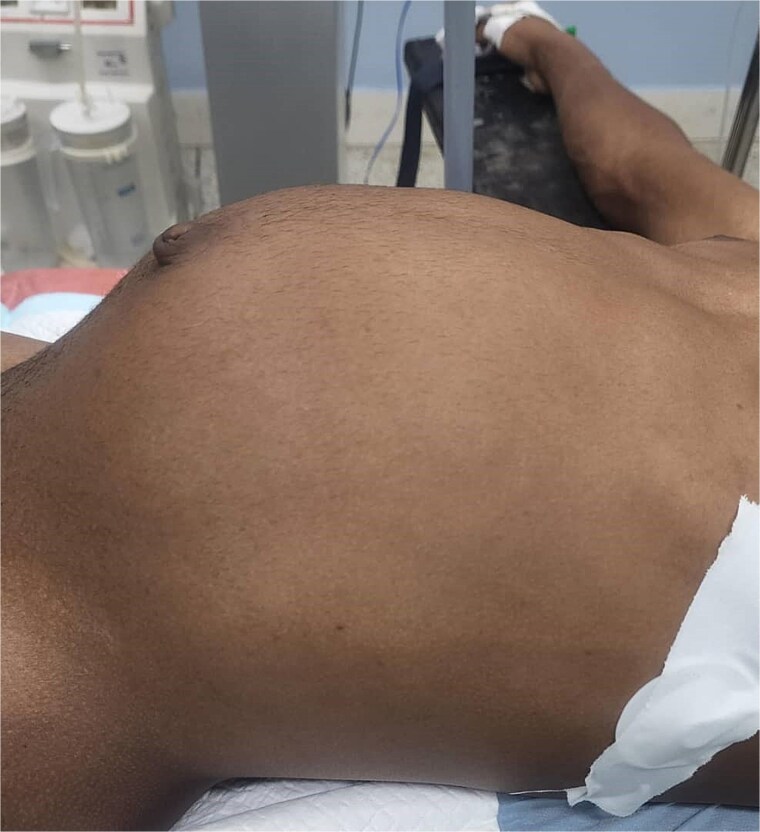
A large, palpable smooth mass measuring ~20 cm in diameter.

On admission, physical examination revealed a distended abdomen, particularly in the upper half. A large, smooth mass measuring ~20 cm in diameter was palpable, occupying the right and left hypochondriac, epigastric, and paraumbilical regions. The mass was non-tender, with no palpable nodules or visible peristalsis (Fig. 1).

**Figure 2:**
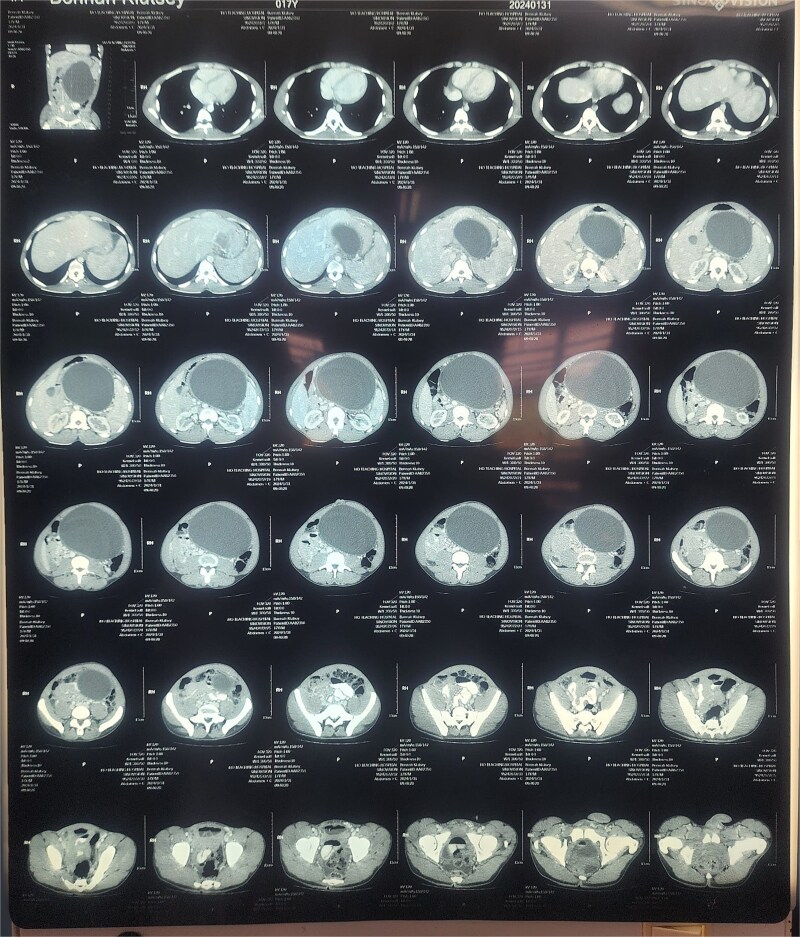
Contrast-enhanced CT scan of the abdomen demonstrating an 18.5 × 10.0 × 13.5 cm well-circumscribed, unilocular pancreatic cyst lesion in the region of the body and tail of the pancreas.

An abdominal ultrasound revealed a giant cystic mass in the epigastrium, extending to the entire upper hemi-abdomen. A subsequent contrast-enhanced computed tomography (CT) scan confirmed a well-circumscribed, thick-walled, unilocular pancreatic cyst lesion in the region of the body and tail, measuring 18.5 × 10.0 × 13.5 cm. The lesion exerted mass effect on the greater curvature of the stomach and displaced the bowel inferiorly. There was no dilatation of the pancreatic duct or calcification (Fig. 2).

**Figure 3:**
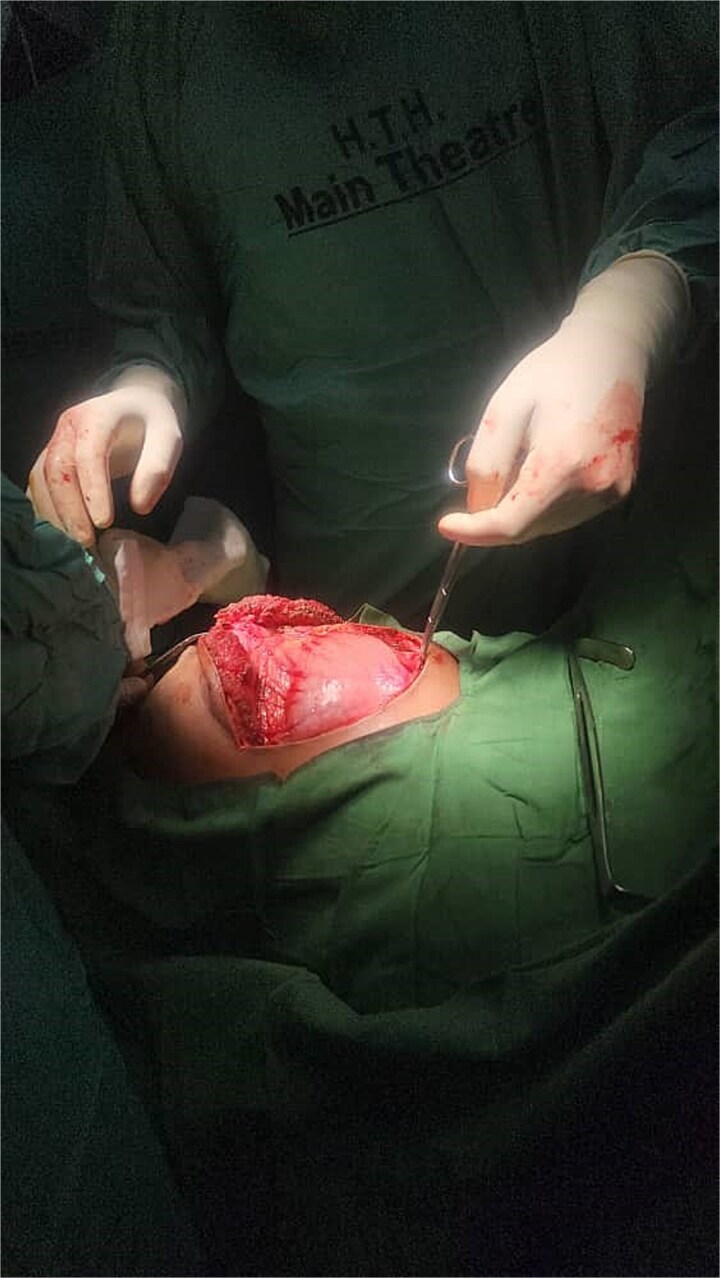
Intraoperative photograph during exploratory laparotomy. The image shows the exposed pancreatic pseudocyst in the upper abdomen, confirming the preoperative diagnosis.

Preoperative investigations revealed hemoglobin level of 8.0 g/dL, necessitating transfusion of two units of blood. Other laboratory results were unremarkable. After this history of upper abdominal trauma, with abdominal distention and imaging finding, concluded a diagnosis of traumatic pancreatic pseudocyst was made. Given the cause, characteristics of the pseudocyst, and maturation of its wall, surgical intervention was deemed necessary. An exploratory laparotomy was performed, confirming the presence of a pancreatic pseudocyst (Fig. 3).

**Figure 4:**
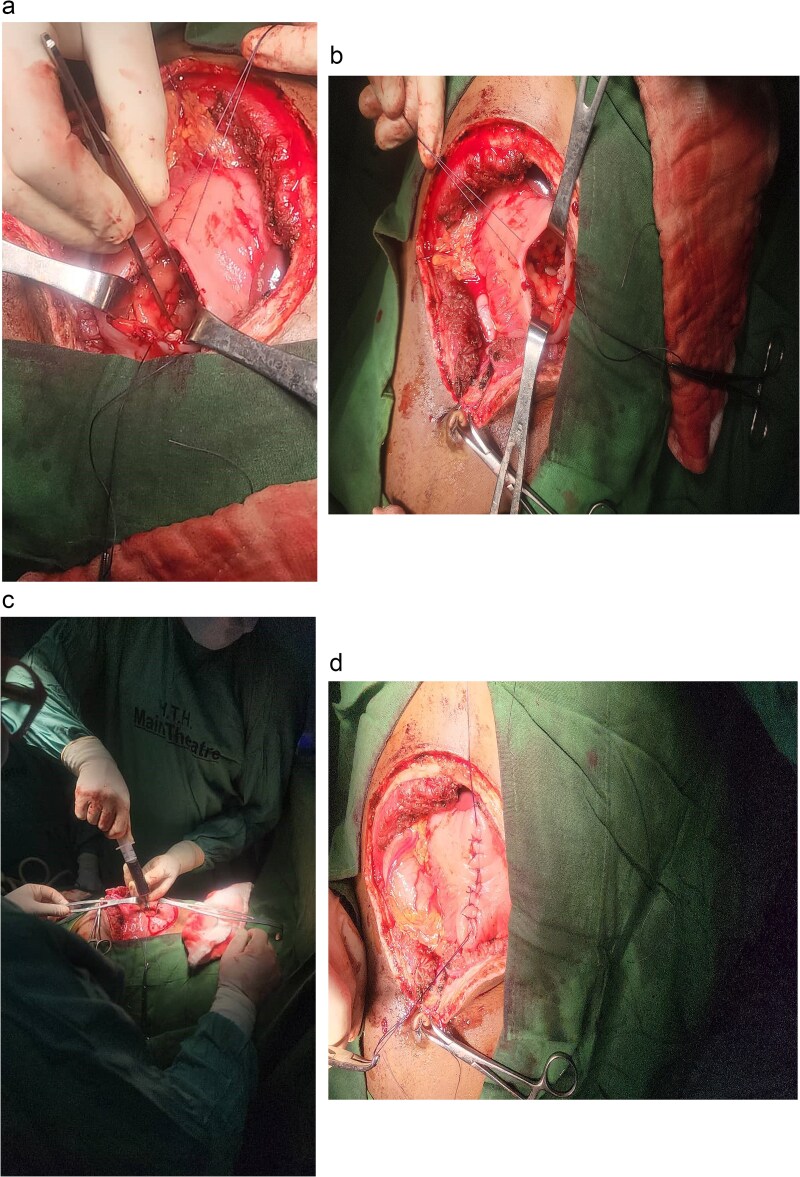
Intraoperative photographs of the transgastric cystogastrostomy procedure. (a) Large cystic mass identified in the lesser sac after midline laparotomy. (b) Anterior gastrotomy exposing the posterior wall of the stomach. (c) Evacuation of ~2850 mL of pseudohemorrhagic fluid from the opened pseudocyst. (d) Completed side-to-side anastomosis between the posterior wall of the stomach and the anterior wall of the pseudocyst, using Vicryl 2-0 sutures in a continuous fashion.

A midline laparotomy incision was made. Upon entering the peritoneal cavity, a large cystic mass was identified in the lesser sac. A trans gastric cystogastrostomy was carried out, involving two incisions in the stomach: one on the anterior wall and another on the posterior wall. The anterior wall of the pseudocyst was opened, and ~2850 mL of pseudo hemorrhagic fluid was evacuated. A wide, tension-free, side-to-side anastomosis was created between the posterior wall of the stomach and the anterior wall of the pseudocyst using Vicryl 2-0 sutures in a continuous fashion. The anterior gastrotomy was then closed in two layers. Hemostasis was ensured, and the abdomen was closed in layers after placing a drain (Fig. 4a–d).

The patient was managed in the surgical intensive care unit for 24 h postoperatively. Pain management included intravenous opioids transitioning to oral analgesics. The nasogastric tube was removed on postoperative day 2, and a liquid diet was initiated. The patient was gradually advanced to a regular diet over the next few days. The drain was removed on postoperative day five.

The patient's immediate postoperative course was uneventful. The fluid culture returned negative results. Histopathological examination of the cyst wall revealed connective tissue with no epithelial lining, granulation tissue, mild lymphocytic infiltration, and numerous macrophages, consistent with the diagnosis of a pancreatic pseudocyst.

**Figure 5:**
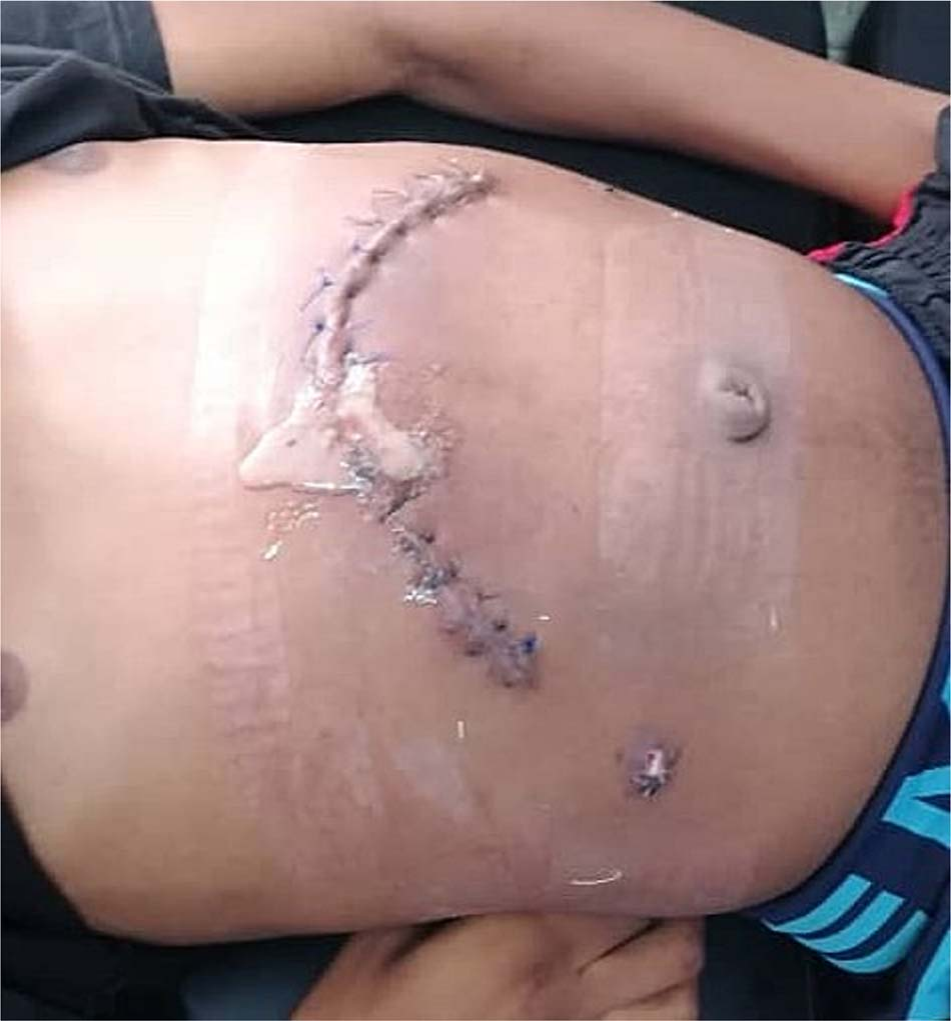
Surgical site at 11 days post-surgery, showing local abscess formation indicative of surgical site infection.

However, 11 days post-surgery, the patient developed a surgical site infection presenting as a local abscess. This complication was treated with local wound care and appropriate antibiotics. After 10 additional days, the wound had cleared. A follow-up abdominal ultrasound showed significant changes in the pancreas, indicating resolution of the pseudocyst (Fig. 5).

**Figure 6:**
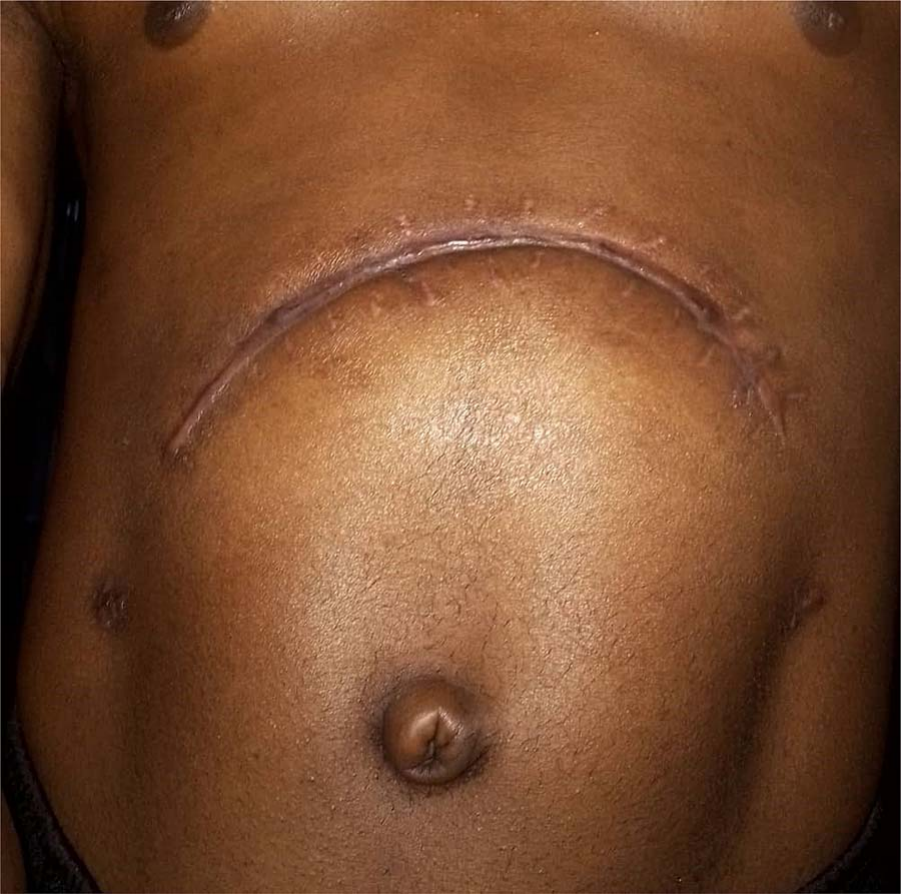
An image of the patient achieving full recovery.

Four weeks after the complication, the patient returned for a review in good general condition and showed progress in recovering his physical status. Another 4 weeks later, the patient had achieved complete recovery. A further follow up with repeat CT is planned at 6 months (Fig. 6).

## Discussion

Giant pancreatic pseudocysts, particularly those resulting from trauma in adolescents, are extremely rare. A literature review revealed only a handful of similar cases in this age group [7]. The choice of cystogastrostomy in this case was based on the size and location of the pseudocyst, as well as the available resources in our facility. While endoscopic and laparoscopic approaches are preferred when available due to their minimally invasive nature [8], open surgical techniques remain viable options in resource-limited settings.

The successful outcome in this case aligns with previous reports suggesting that cystogastrostomy is an effective treatment for large pancreatic pseudocysts [9]. However, the postoperative surgical site infection highlights the potential complications associated with open procedures and the importance of meticulous surgical technique and postoperative care.

## Conclusion

This case demonstrates the successful management of a giant traumatic pancreatic pseudocyst in an adolescent through open cystogastrostomy. It emphasizes the importance of considering this diagnosis in young trauma patients and illustrates the potential for good outcomes with appropriate surgical management and postoperative care, even in resource-limited settings.
